# Acute Bacterial Infections and Longitudinal Risk of Readmissions and Mortality in Patients Hospitalized with Heart Failure

**DOI:** 10.3390/jcm11030740

**Published:** 2022-01-29

**Authors:** Tien M. H. Ng, Esther E. Oh, Yuna H. Bae-Shaaw, Emi Minejima, Geoffrey Joyce

**Affiliations:** 1 Department of Clinical Pharmacy and Medicine, University of Southern California, Los Angeles, CA 90089-9121, USA; 2 Department of Clinical Pharmacy, University of Southern California, Los Angeles, CA 90089-9121, USA; estherohs@gmail.com (E.E.O.); minejima@usc.edu (E.M.); 3 Department of Pharmaceutical and Health Economics, University of Southern California, Los Angeles, CA 90089-9121, USA; hyojungb@usc.edu (Y.H.B.-S.); gjoyce@healthpolicy.usc.edu (G.J.)

**Keywords:** heart failure, acute infection, hospitalization, readmission

## Abstract

Aims: Infections are associated with worse short-term outcomes in patients with heart failure (HF). However, acute infections may have lasting pathophysiologic effects that adversely influence HF outcomes after discharge. Our objective was to describe the impact of acute bacterial infections on longitudinal outcomes of patients hospitalized with a primary diagnosis of HF. Methods and Results: This paper is based on a retrospective cohort study of patients hospitalized with a primary diagnosis of HF with or without a secondary diagnosis of acute bacterial infection in Optum Clinformatics DataMart from 2010–2015. Primary outcomes were 30 and 180-day hospital readmissions and mortality, intensive care unit admission, length of hospital stay, and total hospital charge, compared between those with or without an acute infection. Cohorts were compared after inverse probability of treatment weighting. Multivariable logistic regression was used to examine relationship to outcomes. Of 121,783 patients hospitalized with a primary diagnosis of HF, 27,947 (23%) had a diagnosis of acute infection. After weighting, 30-day hospital readmissions [17.1% vs. 15.7%, OR 1.11 (1.07–1.15), *p* < 0.001] and 180-day hospital readmissions [39.6% vs. 38.7%, OR 1.04 (1.01–1.07), *p* = 0.006] were modestly greater in those with an acute infection versus those without. Thirty-day [5.5% vs. 4.3%, OR 1.29 (1.21–1.38), *p* < 0.001] and 180-day mortality [10.7% vs. 9.4%, OR 1.16 (1.11–1.22), *p* < 0.001], length of stay (7.1 ± 7.0 days vs. 5.7 ± 5.8 days, *p* < 0.001), and total hospital charges (USD 62,200 ± 770 vs. USD 51,100 ± 436, *p* < 0.001) were higher in patients with an infection. Conclusions: The development of an acute bacterial infection in patients hospitalized for HF was associated with an increase in morbidity and mortality after discharge.

## 1. Introduction

Heart failure (HF) remains a substantial health care challenge, with the majority of costs due to direct medical expenditure, specifically hospitalizations. Despite efforts and legislation aimed at optimizing guideline-directed in-hospital medical therapies and improving transitions of care, 30-day readmission rates remain as high as 50%, with half due to non-cardiovascular conditions [1,2]. Three-quarters of these early readmissions are considered preventable, including non-adherence to diet and medical regimens, and poor control of comorbidities [3]. Thus, investigation of factors that result in high readmission rates carry paramount clinical and economic significance. The presence of infections in hospitalized patients portends poorer short-term in-hospital outcomes. The association between acute infections, the development of acute coronary syndromes, and increased morbidity and mortality from cardiovascular causes is also recognized [4,5]. However, little is known about the influence of acute bacterial infections on long-term outcomes of patients with HF, despite there being clear links to pathophysiologic processes that could worsen the progression of HF [6,7,8]. This is especially pertinent in the times of the COVID-19 pandemic and research suggesting potential long-lasting effects on cardiac function. A recent longitudinal analysis of patients with new-onset HF identified infections and respiratory conditions as the two most important causes of non-cardiovascular mortality in these individuals [9]. Patients with HF are at increased risk for infection-related admissions and 30-day mortality compared to patients without HF [10,11,12], with infections commonly the direct cause of death [13]. However, after discharge, the impact of acute infections and their management on the clinical and economic outcomes of patients hospitalized primarily for HF has not been systematically assessed. In addition, the characterization of the type of infections that affect patients hospitalized for HF is limited. Describing the epidemiology of infectious complications, especially bacterial infections, and their impact on outcomes is important to understanding how this comorbidity could be targeted to further improve HF outcomes [14]. This study aims to describe the epidemiology of acute infections, specifically bacterial pathogens, as these represent the most common etiology for infected patients hospitalized for HF [15,16], and to evaluate their associations to longitudinal HF outcomes.

## 2. Methods

This retrospective cohort study utilized pre-existing de-identified data on adult patients ≥ 18 years enrolled in the Optum Clinformatics DataMart, which covers 14 million privately insured and Medicare Advantage beneficiaries, from 1 January 2010 to 30 June 2015. The de-identified Clinformatics^®^ Data Mart (OptumInsight, Eden Prairie, MN, USA) is a large-scale dataset of patients with both medical and pharmacy coverage from a large commercial insurer in the United States. All adult patients hospitalized with a primary diagnosis of HF with or without a secondary diagnosis of an acute bacterial infection during the study period were included in the analysis. All diagnoses were identified by ICD-9 codes (see Appendix A and Appendix B). Patients were excluded if they were <18 years of age, pregnant, incarcerated, had <6 months of follow-up data after index hospital admission, or had infection listed as the primary diagnosis. Patients were categorized into two separate cohorts for comparison: (1) hospitalized patients with HF with a secondary diagnosis of acute infection, and (2) hospitalized patients with HF without an acute infection diagnosis. Patient data were analyzed from index hospitalization to 6 months after discharge. The study was approved by the institutional review board of the University of Southern California (HS-18-00104).

The main outcomes of interest were 30- and 180-day hospital readmissions and mortality (after discharge), length of hospital stay, admission to the intensive care unit (ICU), and total hospital charges. In addition, the types of acute infections and etiology of hospital readmissions (primary diagnosis listed for hospital re-admission) were ascertained. 

Enrollment data on patient demographics (age, gender, marital status) were linked to longitudinal claims capturing admission and discharge dates, discharge to long-term care (LTC), diagnosis of HF (Appendix A), type of acute infection (Appendix B), prescription medications 90 days before and after index hospitalization according to generic drug name, Elixhauser Comorbidity Index, and chronic comorbid diagnoses. The primary diagnoses during hospital readmissions were also obtained. 

As a reflection of the large sample size, at baseline, almost all patient characteristics were statistically significantly different between the cohorts of patients with HF with or without infection. To minimize the effect of these competing differences on the influence of the presence of infection, inverse probability of treatment weighting (IPTW) was applied. The sample with IPTW was weighed to adjust for the propensity score, removing the bias associated with differences in the observed covariates in two groups [17]. The propensity score for infection was estimated using logistic regression with relevant covariates (age, gender, discharge to LTC, Elixhauser Comorbidity Index, and comorbidity). The propensity score (*p*) was then used to weigh the observations in each comparison group so that the patients with infection were given weight of 1/*p* and the patients without infection were given weight of 1/(1 − *p*). Elixhauser Comorbidity Index is a well-validated scoring system to characterize the risk of in-hospital mortality using 31 comorbid conditions and a scoring system of −7 to +12, with higher score indicating greater risk of mortality [18]. The primary and secondary outcomes were measured as both categorical variables (frequencies with percentages) and continuous variables [means with standard deviations (SD)]. Continuous and categorical variables were compared between the two cohorts using the Student’s *t*-test and the chi-squared test, respectively. Multivariable logistic regression was used after the weighting adjustment. A *p*-value of <0.05 was considered statistically significant and 95% confidence intervals were reported for all odds ratios. Statistical analyses were performed using SAS, version 9.4 (SAS Institute, Cary, NC, USA). 

A secondary exploratory analysis was also conducted to determine whether any patient characteristics could independently predict increased risk for acute infections. All subjects in the original non-matched cohort were included. Independent variables evaluated using univariable logistic regression with significance level *p*-value of 0.1 were age, gender, Elixhauser Comorbidity Index, and comorbid conditions (including diabetes mellitus, hypertension, ischemic heart disease, acute or chronic renal failure, obesity, and anemia), cigarette smoking, and statin therapy within 120 days prior to hospitalization. These variables were selected based on available evidence suggesting association with HF outcomes and the hypothetical obtainability of information from the database. Based on the univariable regression results, age, gender, acute renal failure, ischemic heart disease, Elixhauser Comorbidity Index, anemia, obesity, statin therapy prior to hospitalization, and cigarette smoking were further evaluated using a multivariable logistic regression model. The variables were entered in a stepwise manner. Odds ratio and 95% confidence interval (CI) were estimated for the 8 predictive variables in the final model with respect to the event of acute infection during hospitalization. 

## 3. Results

A total of 121,882 patients had an index hospitalization with a primary diagnosis of HF. After excluding 99 patients, due to exclusion criteria or missing data, and weighing (IPTW), 27,912 patients (23%) with HF as a primary diagnosis also had an acute infection secondary diagnosis, while 93,855 patients (77%) did not. A CONSORT diagram illustrates who was included and excluded in Supplemental Figure S1. Baseline characteristics before and after IPTW are reported in Table 1. The mean age of weighted cohorts was 74.5 years, 50% female, 22% discharged to LTC, and mean Elixhauser Comorbidity Index was 4.2. For cardiac comorbidities, 80% had hypertension, 56% had dyslipidemia, 24% had coronary artery disease, and 45% had arrhythmias (any arrhythmia history), whereas for non-cardiac comorbid conditions, 45% had diabetes, 38% had respiratory disorder, and 37% had anemia. 

The major acute infections as secondary diagnoses in patients with HF were commonly identified as pneumonia (60.7%), urinary tract infection (29.9%), or cellulitis (8.7%), while the incidence of sepsis (1.0%) and septic shock (0.7%) was uncommon. Acute infections by type are outlined in Table 2.

In patients with HF and an acute infection, 30-day readmission occurred modestly but significantly more frequently than in patients without an acute infection [17.1% vs. 15.7%, OR 1.11 (1.07–1.15), *p* < 0.001]. The increased risk for readmission was also evident at 180 days [39.6% vs. 38.7%, OR 1.04 (1.01–1.07), *p* = 0.006]. Mortality (after discharge) was greater in patients with HF and an acute infection compared to those without, both at 30 days [5.5% vs. 4.3%, OR 1.29 (1.21–1.38), *p* < 0.001] and 180 days [10.7% vs. 9.4%, OR 1.16 (1.11–1.22), *p* < 0.001]. Hospital readmission and mortality results are outlined in Table 3. The inclusion of variables for state-level education and median household income did not substantially change the ORs related to re-hospitalizations and mortality from the original model.

Since pneumonia and urinary tract infections comprised the majority of the infections, we repeated the outcomes analyses based on these subgroups. When infection was limited to pneumonia, all clinical outcomes remained significantly greater in patients with HF and pneumonia compared to those without infection, except for 180-day readmissions. When infection was limited to urinary tract infection, all clinical outcomes remained greater as well, although the need for ICU admission was not different. Subgroup results are provided in Supplemental Table S1. 

Patients with HF and an acute infection were more likely to be admitted to an ICU compared to those without an acute infection [37.1% vs. 33.8%, OR 1.16 (1.13–1.19), *p* < 0.001]. The length of hospital stay was longer in those with an acute infection, with an absolute difference of 1.4 hospital days. Total hospital charge was also greater in those with an acute infection (USD 62,200 ± 770 vs. 51,100 ± 436, *p* < 0.001), an absolute cost difference of about USD 11,000. For patients readmitted within 30 days, the most frequent primary diagnoses were HF, infection, and acute kidney failure for both cohorts, although numeric differences in the absolute frequency of each diagnosis existed. The ten most frequent primary diagnoses for 30-day readmissions are outlined in Table 4. 

For the exploratory predictors of infection analysis using the unweighted data, patients with HF who developed acute infections were more likely to be older in age (mean 76.7 y vs. 73.8 y), female (57% vs. 47%), have acute renal failure (34.9% vs. 29.5%), and have anemia (40.1% vs. 35.6%) (*p* < 0.0001). Upon multivariable analysis, the significant predictors for increased risk of infection were female gender and acute renal failure, while a history of ischemic heart disease, obesity, and statin therapy was associated with a lower risk of infection. Predictors for acute infection are presented in Supplemental Table S2.

## 4. Discussion

Modifiable comorbid conditions are recognized as significant predictors of outcomes in patients with HF; however, there are limited data related to longitudinal influence of acute infections. This study examined the morbidity and mortality after discharge in patients hospitalized with a primary diagnosis of HF who were also diagnosed with an acute bacterial infection using data from a large national claims database. In the current study, 23% of patients hospitalized primarily for HF developed an acute bacterial infection. The development of an acute infection was associated with an elevated risk for poor clinical and economic outcomes. After propensity matching with IPTW to balance the groups, the odds of 30-day re-hospitalization and 30-day mortality were 10% and 30% higher, respectively, compared to patients who did not develop an acute infection. The increased risk was sustained to 180 days, although slightly attenuated. The development of an acute infection was also associated with a higher proportion of ICU utilization, an increase in mean hospital length of stay by 1.4 days, and higher total hospital charges.

This study represents the largest examination of the prevalence and impact of acute infections in a large U.S. population with a primary admission diagnosis of HF. A previous study in Israel including over 9000 older adults with HF reported 38% of all hospital admissions during a 9-year study period were due to infections [10]. Patients with infection-related hospital admissions had an increase in 6-month and annual readmission rates as well as 30-day and 1-year mortality compared to non-infected patients [10]. A single-center study of 260 patients at a tertiary university hospital in Brazil found that 45.8% of patients developed an acute infection during HF hospitalization, which was associated with increased in-hospital mortality (26.9% vs. 17%, *p* = 0.05), but a decrease in mortality after discharge (11.5% vs. 22%, *p* = 0.046) [19]. However, confounding risk factors for increased in-hospital mortality, such as renal failure, were not accounted for, and it was unknown when mortality after discharge occurred. A recent prospective cohort study of 711 patients with HF in the United Kingdom (UK) identified infection as the cause for readmission in 25% of cases, along with worse survival post discharge [11]. Importantly, cardiovascular causes still accounted for a larger percentage of readmissions (39% overall with 14% due to acute decompensated heart failure) which is in agreement with our results. Infection-related re-hospitalizations were also found in another prospective cohort to independently predict poorer survival [12].

In the current study, an overall 30-day hospital readmission rate of approximately 17% was observed, which is slightly less than the estimated 20–25% risk of hospital readmission reported in patients with generalized HF [20,21,22]. The observed 30-day mortality rate was 5.5% in our cohort with HF and acute infection and 4.3% in those without infection, which is consistent with prior studies that report a 30-day mortality range of 2–20% [10]. There appeared to be an attenuation of re-hospitalization risk with infection based on the 180-day hospitalization odds ratios. This likely reflects other factors which contribute to the outcomes of patients at 180 days, possibly diluting the observed effect or association of the acute infection to 30-day outcomes. In a recent post hoc analysis utilizing clinical trial data of patients with HF, the cumulative incidence of clinical outcomes after hospitalization for pneumonia also attenuated over time [23].

The results have potentially important clinical and policy implications. It has been suggested that 75% of early readmissions are largely preventable and, thus, an area of focus by the U.S. Center for Medicare and Medicaid Services in reducing the national burden of HF costs [1]. Thirty-day performance measures are closely monitored in the U.S., as outcomes during this timeframe are thought to reflect in-hospital HF treatment and adequate transitions of care [1]. Our results indicate that hospitalized patients with HF who experience a complication of acute infection had increased risk of 30-day readmissions and mortality, suggesting that procedures and pathways to optimize infection outcomes should be applied specifically to HF. A recent longitudinal analysis of 86,000 individuals with incident HF also specifically identified infections as an important opportunity to positively impact HF prognosis [9]. The prevention of hospital-acquired infections, early detection of infection using procalcitonin, early initiation of optimal antibiotics, and adequate duration of therapy may prevent and reduce risk of hospital readmissions in patients with HF [14,16,24,25]. The data for procalcitonin for guiding antibiotic decision making remains mixed, but has been more consistently shown to aid in antibiotic de-escalation or discontinuation [26,27]. In the HF population, a procalcitonin-guided approach to early antibiotic initiation failed to improve outcomes in a multicenter study [28]; however, it may be useful in conjunction with other signs and symptoms of inflammation from infection. In a large multi-center U.S. registry study of adults 65 years and older, in-hospital worsening of HF was directly associated with increased mortality, hospital readmissions, and Medicare payments at 30 days [22]. Improving the quality of care during hospitalizations remains an important aspect of HF management and may reduce costs arising from future readmissions.

The association between acute infections and poorer outcomes is plausible, both clinically and pathophysiologically. In our analysis of predictors of acute infections, older age, female gender, acute renal failure, and anemia were all associated with increased risk. Renal failure and anemia are well recognized for their association to poorer prognosis in HF [22,29,30], and thus it could be hypothesized that the link between infections and worse outcomes is an epiphenomenon. However, the lack of predictive value of the Elixhauser comorbidity score for infection and the IPTW analysis that eliminated baseline differences in these covariates support the independent risks associated with acute infection. Pathophysiologically, infections and HF share at least two common pathways—inflammation and neurohormonal activation [31,32]. Severe infections that require hospitalization have been identified as a potential trigger of the inflammatory process and a risk factor for coronary artery disease. In addition, the risk of subsequent and delayed cardiovascular disease is reported to be six-fold during the first year after infection [33,34]. Recently, a 13-year study looking at 1.2 million patients admitted to UK hospitals reported that patients with a prior infection had higher incidence of ischemic heart disease and stroke, which was associated with significantly higher mortality than the general population [35]. This suggests that infections cause local and systemic inflammation and have procoagulant properties that can contribute to the cardiovascular disease process. Infection-induced inflammation may also impair inotropy and contribute to adverse cardiac remodeling, negatively influencing patients with HF [36,37,38]. Inflammation and immune system function are also closely tied to activation of the sympathetic nervous system, and the counter-regulation of these systems may be an important mechanism of worse prognosis in patients with HF who develop infections [31,39]. Our findings of an inverse association between history of ischemic heart disease, obesity, and statin use to risk of infection was not expected. However, one potential explanation is that our analysis first captured re-hospitalization and, since the more common reason for hospitalization in these patients was cardiovascular and not infectious, it may appear as an observed “protective” association. Two recent analyses examining reasons for hospitalizations post-myocardial infarction also found that the majority were cardiac [40,41]. Statins, which exert anti-inflammatory and possible immunomodulatory effects, may lower the risk of infections although it remains an area of controversy [42,43,44].

Literature characterizing infections in patients with HF is emerging. The current study reflects a large national diverse population, and the most common acute infections in patients hospitalized with HF were pneumonia and urinary tract infections. Both of these infection subgroups had an increase in 30-day mortality and hospital readmissions. These findings are consistent with results from a large UK database study where respiratory and urinary tract infections were independently associated with an increase in myocardial infarction and stroke during the 3-day post-infectious period [45]. Respiratory infections in particular have also been independently associated with higher in-hospital mortality in patients with HF and mentioned in large registries and observational cohort studies as the most common type of infection in hospitalized HF patients [12,36,46,47]. Another study found that 52.6% of patients hospitalized with HF developed a lower respiratory tract infection and 15.7% developed a urinary tract infection, further supporting these infections as an important area for future research [10]. 

Data describing specific prevention methods of hospital-acquired infections in the HF population is lacking. However, general recommendations to minimize infection risk for the most common hospital-acquired infections are likely applicable to the patient hospitalized for HF. This includes optimal management of comorbidities, eliminating catheters or lines as soon as possible, appropriate ventilator care for intubated patients, discontinuing unnecessary stress-ulcer prophylaxis and antibiotics to prevent Clostridium difficile infection, appropriate hand hygiene and patient isolation precautions, and ensuring that vaccinations are up to date.

There are several limitations to our study that deserve mention. First, inherent limitations of utilizing coded data for diagnoses are well described and apply to our analyses. However, the database has been used for similar type of epidemiologic studies that have been well validated. In retrospect, some ICD-9 subcodes that could indicate bacterial infections were omitted from the original analysis. When outcomes were re-analyzed using a revised ICD-9 code set which expanded potential capture of intestinal infections (008.x), pneumonia (482.x), more specific codes for septicemia from only bacterial organisms (038.x), unspecified site bacterial infections (041.x), meningitis (320.x), bacterial endocarditis (421.x), peritonitis (567.x), liver abscess (572.x), and some others, the odds ratios were similar, confirming the association of increased risk. Importantly, all these broad infection sources were included in the original set of ICD-9 codes. Due to the lack of availability of in-hospital data, medication use at the time of admission and discharge was estimated by evaluating prescription charges 90 days before and after the index hospitalization. Lastly, the Optum Clinformatics database has limited patient-level clinical data, so we were unable to identify specific HF characteristics and biomarkers (e.g., left ventricular ejection fraction, blood pressure, laboratory values, etc.) with prognostic importance to further risk-stratify and control these differences in our analyses.

Further study is needed to continue to advance our understanding of how infections intersect with HF outcomes. Since we did not evaluate non-bacterial acute infections (i.e., fungal or viral infections), it would be valuable to determine specific pathogens that are more commonly seen in patients hospitalized with HF. This could potentially further validate studies that support regular use of influenza and pneumococcal vaccinations in patients with HF, since this is a seemingly cost-effective intervention that could improve quality of life and clinical outcomes [36,48]. Lastly, the establishment of acute infections as a modifiable risk factor for HF outcomes would support the added value of adequate antimicrobial management and provide a basis for future studies to evaluate the impact of these interventions on HF outcomes. 

In conclusion, acute bacterial infections are a common occurrence in patients hospitalized with HF and are associated with an increase in longitudinal hospital readmissions and mortality after discharge. Patients hospitalized with HF who develop an infection also have increased hospital length of stay and total hospital charge.

## Figures and Tables

**Table 1:** Baseline characteristics before and after inverse probability of treatment weighting.

Characteristics	Unweighted			Weighted		
	HF with Infection (N = 27,947)	HF without Infection (N = 93,836)	*p*-Value	HF with Infection (N = 27,912)	HF without Infection (N = 93,855)	*p*-Value
Age, mean y (SD)	76.7 (10.1)	73.8 (11.5)	<0.001	74.5 (11.4)	74.5 (11.3)	0.8
Female sex, %	57	47	<0.001	50	50	0.253
Discharged to LTC, %	34	18	<0.001	22	22	0.805
Elixhauser Comorbidity Index, mean (SD)	3.95 (5.64)	4.26 (6.34)	<0.001	4.22 (5.85)	4.20 (6.28)	0.508
*Comorbid Conditions* (%)						
Diabetes Type 1 and 2	45	46	0.026	46	45	0.572
Hypertension	80	80	0.087	80	80	0.711
Hyperlipidemia	52	57	<0.001	56	56	0.914
Acute Myocardial Infarction	1.2	1.2	0.379	1.2	1.2	0.911
Ischemic Heart Disease	20	26	<0.001	24	24	0.516
Cerebral Ischemia	12	10	<0.001	11	11	0.983
Intracranial Hemorrhage	0.7	0.5	0.007	0.6	0.5	0.48
Arrhythmia	48	45	<0.001	45	45	0.894
Bronchitis	42	37	<0.001	38	38	0.331
Anemia	40	36	<0.001	37	37	0.756
Chronic Renal Failure	38	39	0.052	39	39	0.85
Acute Renal Failure	35	30	<0.001	31	31	0.857
Obesity	15	17	<0.001	17	16	0.407
Sleep Apnea	12	15	<0.001	14	14	0.525
Smoking	9.1	11	<0.001	10	10	0.969
Renal Failure	9.2	8.3	<0.001	8.5	8.5	0.987
Liver Disease	1.8	1.6	0.083	1.8	1.6	0.066
Alcohol	1	1.3	<0.001	1.2	1.3	0.286
Cancer	0.9	0.8	0.008	0.9	0.8	0.004
Illicit Drugs	0.2	0.3	<0.001	0.3	0.3	0.059
Emphysema	0	0	0.365	0	0	0.761
Asthma	0	0	0.2	0	0	0.436
*Admission Medications* (%)						
ACE Inhibitor	55	57	<0.001	56	56	0.724
ARB	29	31	<0.001	30	30	0.588
MRA/thiazide	20	22	<0.001	21	22	0.493
Loop Diuretic	47	46	0.058	46	46	0.667
Digoxin	8.9	9	0.792	8.9	9	0.807
Nitrate	17	18	<0.001	18	18	0.582
Beta Blocker	51	53	<0.001	52	52	0.812
Calcium Channel Blocker	28	25	<0.001	26	26	0.804
Statin	40	41	0.003	41	41	0.782
Antiplatelet	14	15	<0.001	14	14	0.858
Anticoagulant	21	21	0.441	21	21	0.838
Antiarrhythmic	7	7.6	<0.001	7.4	7.5	0.73
*Discharge Medications* (%)						
ACE Inhibitor	57	65	<0.001	63	63	0.963
ARB	30	37	<0.001	35	35	0.608
MRA/thiazide	20	27	<0.001	25	25	0.857
Loop Diuretic	61	67	<0.001	66	66	0.587
Digoxin	11	12	<0.001	12	12	0.652
Nitrate	20	24	<0.001	23	23	0.723
Beta Blocker	55	63	<0.001	61	61	0.951
Calcium Channel Blocker	23	23	0.154	23	23	0.926
Statin	38	44	<0.001	42	42	0.993
Antiplatelet	13	15	<0.001	15	15	0.943
Anticoagulant	22	24	<0.001	24	24	0.704
Antiarrhythmic	8.3	9.8	<0.001	9.4	9.4	0.772

ACE inhibitor = angiotensin converting enzyme inhibitor, ARB = angiotensin receptor blocker, HF = heart failure, LTC = long-term care facility, MRA = mineralocorticoid receptor antagonist, SD = standard deviation.

**Table 2:** Type of acute bacterial infection.

ICD-9 Code	Infection Type	HF with Infection N (%)
486	Pneumonia	15,953 (57.1)
599.0	Urinary tract infection, site not specified	8346 (29.9)
682.6	Cellulitis and abscess of leg, except foot Abscess of lower limb, cellulitis of lower limb	2438 (8.7)
507.0	Pneumonia due to inhalation of food or vomitus	1103 (3.6)
995.91	Sepsis	274 (1.0)
995.92	Severe Sepsis	331 (1.2)
790.7	Bacteremia	357 (1.3)
785.52	Septic Shock	181 (0.7)
N/A	Others	1062 (3.8)
N/A	Missing	196 (0.7)

**Table 3:** Inverse probability of treatment weighted primary and secondary outcomes and multivariable regression.

	HF with Infection (N = 27,912)	HF without Infection (N = 93,855)	*p*-Value	Odds Ratio	95% CI
30-day readmission, *n* (%)	4745 (17.1)	14,829 (15.7)	<0.001	1.11	1.07–1.15
180-day readmission, *n* (%)	11,025 (39.6)	36,040 (38.7)	0.006	1.04	1.01–1.07
30-day mortality, *n* (%)	1535 (5.5)	4036 (4.3)	<0.001	1.29	1.21–1.38
180-day mortality, *n* (%)	2987 (10.7)	8822 (9.4)	<0.001	1.16	1.11–1.22
ICU admission, *n* (%)	10,355 (37.1)	31,722 (33.8)	<0.001	1.16	1.13–1.19
Length of stay, days (SD)	7.1 (7.0)	5.7 (5.8)	<0.001	--	--
Hospital charge, USD (SEM)	62,200 (770)	51,100 (436)	<0.001	--	--

CI = confidence interval, SD = standard deviation, SEM = standard error of the mean.

**Table 4:** Ten most frequent primary diagnoses at 30-day readmission.

	HF with Infection			HF without Infection		
	ICD-9	Description	%	ICD-9	Description	%
1	428.33	Acute on chronic diastolic HF	7.7	428.23	Acute on chronic systolic HF	10.6
2	428.23	Acute on chronic systolic HF	7.5	428.33	Acute on chronic diastolic HF	7.0
3	038.9	Unspecified septicemia	5.6	428.0	Congestive HF	5.8
4	584.9	Acute kidney failure	5.3	584.9	Acute kidney failure	5.3
5	428.0	Congestive HF	4.9	428.43	Acute on chronic systolic and diastolic HF	3.3
6	486	Pneumonia	3.9	038.9	Unspecified septicemia	3.1
7	428.43	Acute on chronic systolic and diastolic HF	3.2	427.31	Atrial fibrillation	2.7
8	491.21	Acute obstructive bronchitis	2.4	486	Pneumonia	2.2
9	518.81	Acute respiratory failure	2.3	491.21	Acute obstructive bronchitis	2.0
10	427.31	Atrial fibrillation	2.2	404.91	Hypertensive heart and CKD	1.7

CKD = chronic kidney disease; HF = heart failure.

## Data Availability

Restrictions apply to the availability of these data. The data were available under an institutional data use agreement with OptumInsight, Eden Prairie, MN.

## Supplementary Materials

The following supporting information can be downloaded at: https://www.mdpi.com/article/10.3390/jcm11030740/s1, Figure S1. Assessment for Study Eligibility: Diagram of eligibility criteria for inclusion into (A) heart failure with infection or (B) heart failure without infection cohorts. Table S1. Inverse Probability of Treatment Weighted Primary and Secondary Outcomes, and Multivariable Regression based on Pneumonia and Urinary Tract Infection Subgroups. Table S2. Predictors of Acute Infection in Patients with Heart Failure.

## Appendix A

**Table A1:** ICD-9 Codes for Heart Failure.

402.01	Hypertensive heart disease, malignant, with heart failure MAL HYPERT HRT DIS W HF
402.11	Hypertensive heart disease, benign, with heart failure BENIGN HYP HT DIS W HF
402.91	Hypertensive heart disease, unspecified, with heart failure HYP HT DIS NOS W HT FAIL
404.01	Hypertensive heart and chronic kidney disease, malignant, with heart failure and with chronic kidney disease stage I through stage IV, or unspecified MAL HYP HT/KD I-IV W HF
404.03	Hypertensive heart and chronic kidney disease, malignant, with heart failure and with chronic kidney disease stage V or end stage renal disease MAL HYP HT/KD STG V W H
404.11	Hypertensive heart and chronic kidney disease, benign, with heart failure and with chronic kidney disease stage I through stage IV, or unspecified BEN HYP HT/KD I-IV W HF
404.13	Hypertensive heart and chronic kidney disease, benign, with heart failure and chronic kidney disease stage V or end stage renal disease BEN HYP HT/KD STG V W HF
404.91	Hypertensive heart and chronic kidney disease, unspecified, with heart failure and with chronic kidney disease stage I through stage IV, or unspecified HYP HT/KD NOS I-IV W HF
404.93	Hypertensive heart and chronic kidney disease, unspecified, with heart failure and chronic kidney disease stage V or end stage renal disease HYP HT/KD NOS ST V W HF
428.0	Congestive heart failure, unspecified CHF NOS
428.1	Left heart failure LEFT HEART FAILURE
428.20	Unspecified systolic heart failure SYSTOLIC HRT FAILURE NOS
428.21	Acute systolic heart failure AC SYSTOLIC HRT FAILURE
428.22	Chronic systolic heart failure CHR SYSTOLIC HRT FAILURE
428.23	Acute on chronic systolic heart failure AC ON CHR SYST HRT FAIL
428.30	Unspecified diastolic heart failure DIASTOLC HRT FAILURE NOS
428.31	Acute diastolic heart failure AC DIASTOLIC HRT FAILURE
428.32	Chronic diastolic heart failure CHR DIASTOLIC HRT FAIL
428.33	Acute on chronic diastolic heart failure AC ON CHR DIAST HRT FAIL
428.40	Unspecified combined systolic and diastolic heart failure SYST/DIAST HRT FAIL NOS
428.41	Acute combined systolic and diastolic heart failure AC SYST/DIASTOL HRT FAIL
428.42	Chronic combined systolic and diastolic heart failure CHR SYST/DIASTL HRT FAIL
428.43	Acute on chronic combined systolic and diastolic heart failure AC/CHR SYST/DIA HRT FAIL
428.9	Heart failure, unspecified HEART FAILURE NOS

## Appendix B

**Table A2:** ICD-9 Codes for Acute Bacterial Infections.

461.9	Acute Sinusitis, Unspecified
462	Acute Pharyngitis
465.9	Acute Upper Respiratory Infections of Unspecified Site
486	Pneumonia, Organism, Unspecified
997.31	Ventilator Associated Pneumonia
507.0	Pneumonia due to inhalation of food or vomitus
513.0	Abscess of Lung
008.45	Intestinal Infection Due to Clostridium Difficile
009.0	Infectious Colitis, Enteritis, and Gastroenteritis
567.22	Peritoneal Abscess
682.6	Cellulitis and Abscess of Leg, Except Foot, Abscess of Lower Limb Cellulitis of Lower Limb
682.9	Cellulitis And Abscess of Unspecified Site, Cutaneous Abscess, Unspecified Cellulitis Of Lower Limb
686.9	Unspecified Local Infection of Skin And Subcutaneous Tissue
728.86	Necrotizing Fasciitis
599.0	Urinary Tract Infection, Site Not Specified
595	Acute Cystitis, Acute Cystitis Without Hematuria, Acute Cystitis With Hematuria
595.9	Unspecified Cystitis, Cystitis, Unspecified Without Hematuria Cystitis, Unspecified With Hematuria
599.0	Urinary Tract Infection, Site Not Specified
590.10	Acute Pyelonephritis without lesion of renal medullary necrosis
590.80	Pyelonephritis, unspecified
136.9	Unspecified Infectious And Parasitic Diseases
322.9	Meningitis, Unspecified
995.91	Sepsis
995.92	Severe Sepsis
785.52	Septic Shock
038	Septicemia
790.7	Bacteremia
999.32	Bloodstream infection due to central venous catheter
996.62	Infection and Inflammatory Reaction Due to Other Vascular Device, Implant, and Graft
996.64	Infection and Inflammatory Reaction due to Indwelling Urinary Catheter
998.59	Other Postoperative Infection
711.0	Pyogenic Arthritis
730.0	Acute Osteomyelitis
324.0	Intracranial Abscess
324.1	Intraspinal Abscess
422.0	Acute Myocarditis in Diseases Classified Elsewhere
420.90	Acute Pericarditis, Unspecified
519.2	Mediastinitis
421.9	Acute Endocarditis, Unspecified
V09.8	Infection with microorganisms resistant to other specified drugs
V09.1	Infection with microorganisms resistant to cephalosporin and other B-lactam antibiotics
041.89	Other Specified Bacterial Infections in Conditions Classified Elsewhere and of Unspecified Site, Other Specified Bacteria

## References

[1] Desai A.S., Stevenson L.W. (2012). Rehospitalization for heart failure: Predict or prevent?. Circulation.

[2] Geppert A., Steiner A., Delle-Karth G., Heinz G., Huber K. (2003). Usefulness of procalcitonin for diagnosing complicating sepsis in patients with cardiogenic shock. Intensive Care Med..

[3] Cheng J.W., Cooke-Ariel H. (2014). Pharmacists’ role in the care of patients with heart failure: Review and future evolution. J. Manag. Care Pharm..

[4] Musher D.M., Abers M., Corrales-Medina V.F., Frank R.C., Hanidziar D. (2019). Acute Infection and Myocardial Infarction. N. Engl. J. Med..

[5] Boehme A.K., Kulick E.R., Canning M., Alvord T., Khaksari B., Omran S., Willey J.Z., Elkind M.S.V. (2018). Infections Increase the Risk of 30-Day Readmissions Among Stroke Survivors. Stroke.

[6] Nagatomo Y., Tang W.H. (2015). Intersections Between Microbiome and Heart Failure: Revisiting the Gut Hypothesis. J. Card Fail..

[7] Frantz S., Falcao-Pires I., Balligand J.-L., Bauersachs J., Brutsaert D., Ciccarelli M., Dawson D., De Windt L.J., Giacca M., Hamdani N. (2018). The innate immune system in chronic cardiomyopathy: A European Society of Cardiology (ESC) scientific statement from the Working Group on Myocardial Function of the ESC. Eur. J. Heart Fail..

[8] Mann D.L. (2015). Innate immunity and the failing heart: The cytokine hypothesis revisited. Circ. Res..

[9] Conrad N., Judge A., Canoy D., Tran J., Pinho-Gomes A.C., Millett E.R.C., Salimi-Khorshidi G., Cleland J.G., McMurray J.J.V., Rahimi K. (2019). Temporal Trends and Patterns in Mortality After Incident Heart Failure: A Longitudinal Analysis of 86000 Individuals. JAMA Cardiol..

[10] Alon D., Stein G.Y., Korenfeld R., Fuchs S. (2013). Predictors and outcomes of infection-related hospital admissions of heart failure patients. PLoS ONE.

[11] Drozd M., Garland E., Walker A.M.N., Slater T.A., Koshy A., Straw S., Gierula J., Paton M., Lowry J., Sapsford R. (2020). Infection-Related Hospitalization in Heart Failure with Reduced Ejection Fraction: A Prospective Observational Cohort Study. Circ. Heart Fail..

[12] Cheng C.-W., Liu M.-H., Wang C.-H. (2020). Predictors of infection-related rehospitalization in heart failure patients and its impact on long-term survival. J. Cardiovasc. Med..

[13] Murad K., Kitzman D.W. (2012). Frailty and multiple comorbidities in the elderly patient with heart failure: Implications for management. Heart Fail. Rev..

[14] Aïssou L., Sorbets E., Lallmahomed E., Goudot F.-X., Pop N., Es-Sebbani S., Benouda L., Nuel G., Meune C. (2018). Prognostic and diagnostic value of elevated serum concentration of procalcitonin in patients with suspected heart failure. A review and meta-analysis. Biomarkers.

[15] Mamic P., Heidenreich P.A., Hedlin H., Tennakoon L., Staudenmayer K.L. (2016). Hospitalized Patients with Heart Failure and Common Bacterial Infections: A Nationwide Analysis of Concomitant Clostridium Difficile Infection Rates and In-Hospital Mortality. J. Card. Fail..

[16] Möckel M., Searle J., Maisel A. (2017). The role of procalcitonin in acute heart failure patients. ESC Heart Fail..

[17] Hirano K., Imbens G.W., Ridder G. (2003). Efficient estimation of average treatment effects using the estimated propensity score. Econometrica.

[18] Moore B.J., White S., Washington R., Coenen N., Elixhauser A. (2017). Identifying Increased Risk of Readmission and In-hospital Mortality Using Hospital Administrative Data: The AHRQ Elixhauser Comorbidity Index. Med. Care.

[19] Cardoso J.N., DEL Carlo C.H., Junior M.T.D.O., Ochiai M.E., Filho R.K., Barretto A.C.P. (2018). Infection in Patients with Decompensated Heart Failure: In-Hospital Mortality and Outcome. Arq. Bras. Cardiol..

[20] Bergethon K.E., Ju C., DeVore A.D., Hardy N.C., Fonarow G.C., Yancy C.W., Heidenreich P.A., Bhatt D.L., Peterson E.D., Hernandez A.F. (2016). Trends in 30-Day Readmission Rates for Patients Hospitalized with Heart Failure: Findings from the Get With The Guidelines-Heart Failure Registry. Circ. Heart Fail..

[21] Blecker S., Paul M., Taksler G., Ogedegbe G., Katz S. (2013). Heart Failure–Associated Hospitalizations in the United States. J. Am. Coll. Cardiol..

[22] DeVore A.D., Hammill B.G., Sharma P.P., Qualls L.G., Mentz R.J., Johnson K.W., Fonarow G.C., Curtis L.H., Hernandez A.F. (2014). In-Hospital Worsening Heart Failure and Associations with Mortality, Readmission, and Healthcare Utilization. J. Am. Heart Assoc..

[23] Shen L., Jhund P.S., Anand I.S., Bhatt A.S., Desai A.S., Maggioni A.P., Martinez F.A., Pfeffer M.A., Rizkala A.R., Rouleau J.L. (2021). Incidence and Outcomes of Pneumonia in Patients with Heart Failure. J. Am. Coll. Cardiol..

[24] Demissei B.G., Cleland J.G., O’Connor C.M., Metra M., Ponikowski P., Teerlink J.R., Davison B., Givertz M.M., Bloomfield D.M., Dittrich H. (2016). Procalcitonin-based indication of bacterial infection identifies high risk acute heart failure patients. Int. J. Cardiol..

[25] Schuetz P., Kutz A., Grolimund E., Haubitz S., Demann D., Vögeli A., Hitz F., Christ-Crain M., Thomann R., Falconnier C. (2014). Excluding infection through procalcitonin testing improves outcomes of congestive heart failure patients presenting with acute respiratory symptoms: Results from the randomized ProHOSP trial. Int. J. Cardiol..

[26] Wussler D., Kozhuharov N., Oliveira M.T., Bossa A., Sabti Z., Nowak A., Murray K., Lavallaz J.D.F.D., Badertscher P., Twerenbold R. (2019). Clinical Utility of Procalcitonin in the Diagnosis of Pneumonia. Clin. Chem..

[27] Banach J., Wołowiec Ł., Rogowicz D., Gackowska L., Kubiszewska I., Gilewski W., Michałkiewicz J., Sinkiewicz W. (2018). Procalcitonin (PCT) Predicts Worse Outcome in Patients with Chronic Heart Failure with Reduced Ejection Fraction (HFrEF). Dis. Markers.

[28] Möckel M., De Boer R.A., Slagman A., Von Haehling S., Schou M., Vollert J.O., Wiemer J.C., Ebmeyer S., Martín-Sánchez F.J., Maisel A.S. (2019). Improve Management of acute heart failure with ProcAlCiTonin in EUrope: Results of the randomized clinical trial IMPACT EU Biomarkers in Cardiology (BIC) 18. Eur. J. Heart Fail..

[29] Ahmed A., Campbell R.C. (2008). Epidemiology of chronic kidney disease in heart failure. Heart Fail. Clin..

[30] Löfman I., Szummer K., Hagerman I., Dahlström U., Lund L.H., Jernberg T. (2016). Prevalence and prognostic impact of kidney disease on heart failure patients. Open Heart.

[31] Eurich D.T., Marrie T.J., Minhas-Sandhu J.K., Majumdar S.R. (2017). Risk of heart failure after community acquired pneumonia: Prospective controlled study with 10 years of follow-up. BMJ.

[32] Kakihana Y., Ito T., Nakahara M., Yamaguchi K., Yasuda T. (2016). Sepsis-induced myocardial dysfunction: Pathophysiology and management. J. Intensive Care.

[33] Bergh C., Fall K., Udumyan R., Sjöqvist H., Fröbert O., Montgomery S. (2017). Severe infections and subsequent delayed cardiovascular disease. Eur. J. Prev. Cardiol..

[34] Roivainen M., Viik-Kajander M., Palosuo T., Toivanen P., Leinonen M., Saikku P., Tenkanen L., Manninen V., Hovi T., Mänttäri M. (2000). Infections, Inflammation, and the Risk of Coronary Heart Disease. Circulation.

[35] Carter P., Uppal H., Bainey K., Potluri R. (2018). Infection is a risk factor for ischemic heart disease and stroke and impacts long-term mortality: Insights utilsing big data from the UK ACALM Registry. J. Am. Coll. Cardiol..

[36] Bhatt A.S., Liang L., DeVore A.D., Fonarow G.C., Solomon S.D., Vardeny O., Yancy C.W., Mentz R.J., Khariton Y., Chan P.S. (2018). Vaccination Trends in Patients with Heart Failure: Insights from Get with the Guidelines-Heart Failure. JACC Heart Fail..

[37] Winklewski P.J., Radkowski M., Demkow U. (2014). Cross-talk between the inflammatory response, sympathetic activation and pulmonary infection in the ischemic stroke. J. Neuroinflamm..

[38] Van Linthout S., Tschope C. (2017). Inflammation—Cause or Consequence of Heart Failure or Both?. Curr. Heart Fail. Rep..

[39] Ng T.M., Toews M.L. (2016). Impaired norepinephrine regulation of monocyte inflammatory cytokine balance in heart failure. World J. Cardiol..

[40] Rymer J.A., Chen A.Y., Thomas L., Fonarow G.C., Peterson E.D., Wang T.Y. (2019). Readmissions after Acute Myocardial Infarction: How Often Do Patients Return to the Discharging Hospital?. J. Am. Heart Assoc..

[41] Wang H., Zhao T., Wei X., Lu H., Lin X. (2019). The prevalence of 30-day readmission after acute myocardial infarction: A systematic review and meta-analysis. Clin. Cardiol..

[42] Nassaji M., Ghorbani R., Afshar R.K. (2015). The Effect of Statins Use on the Risk and Outcome of Acute Bacterial Infections in Adult Patients. J. Clin. Diagn. Res..

[43] Tleyjeh I.M., Kashour T., Hakim F.A., Zimmerman V.A., Erwin P.J., Sutton A.J., Ibrahim T. (2009). Statins for the prevention and treatment of infections: A systematic review and meta-analysis. Arch. Intern. Med..

[44] Van den Hoek H.L., Bos W.J., de Boer A., van de Garde E.M. (2011). Statins and prevention of infections: Systematic review and meta-analysis of data from large randomised placebo controlled trials. BMJ.

[45] Smeeth L., Thomas S.L., Hall A.J., Hubbard R., Farrington C.P., Vallance P. (2004). Risk of Myocardial Infarction and Stroke after Acute Infection or Vaccination. N. Engl. J. Med..

[46] Fonarow G.C., Abraham W.T., Albert N.M., Stough W.G., Gheorghiade M., Greenberg B.H., O’Connor C.M., Pieper K., Sun J.L., Yancy C.W. (2008). Factors Identified as Precipitating Hospital Admissions for Heart Failure and Clinical OutcomesFindings From OPTIMIZE-HF. Arch. Intern. Med..

[47] Ueda T., Kawakami R., Horii M., Sugawara Y., Matsumoto T., Okada S., Nishida T., Soeda T., Okayama S., Somekawa S. (2014). Noncardiovascular Death, Especially Infection, Is a Significant Cause of Death in Elderly Patients with Acutely Decompensated Heart Failure. J. Card. Fail..

[48] Kadoglou N.P.E., Bracke F., Simmers T., Tsiodras S., Parissis J. (2017). Influenza infection and heart failure—vaccination may change heart failure prognosis?. Heart Fail. Rev..

