# Supplementary material for: Acute Bacterial Infections and Longitudinal Risk of Readmissions and Mortality in Patients Hospitalized with Heart Failure
Source: J Clin Med. 2022 Jan 29;11(3):740. doi: 10.3390/jcm11030740 (PMC8836984; doi:10.3390/jcm11030740)
Supplement: Supplementary file 1 [file jcm-11-00740-s001.zip › jcm-1517347-supplementary.pdf]

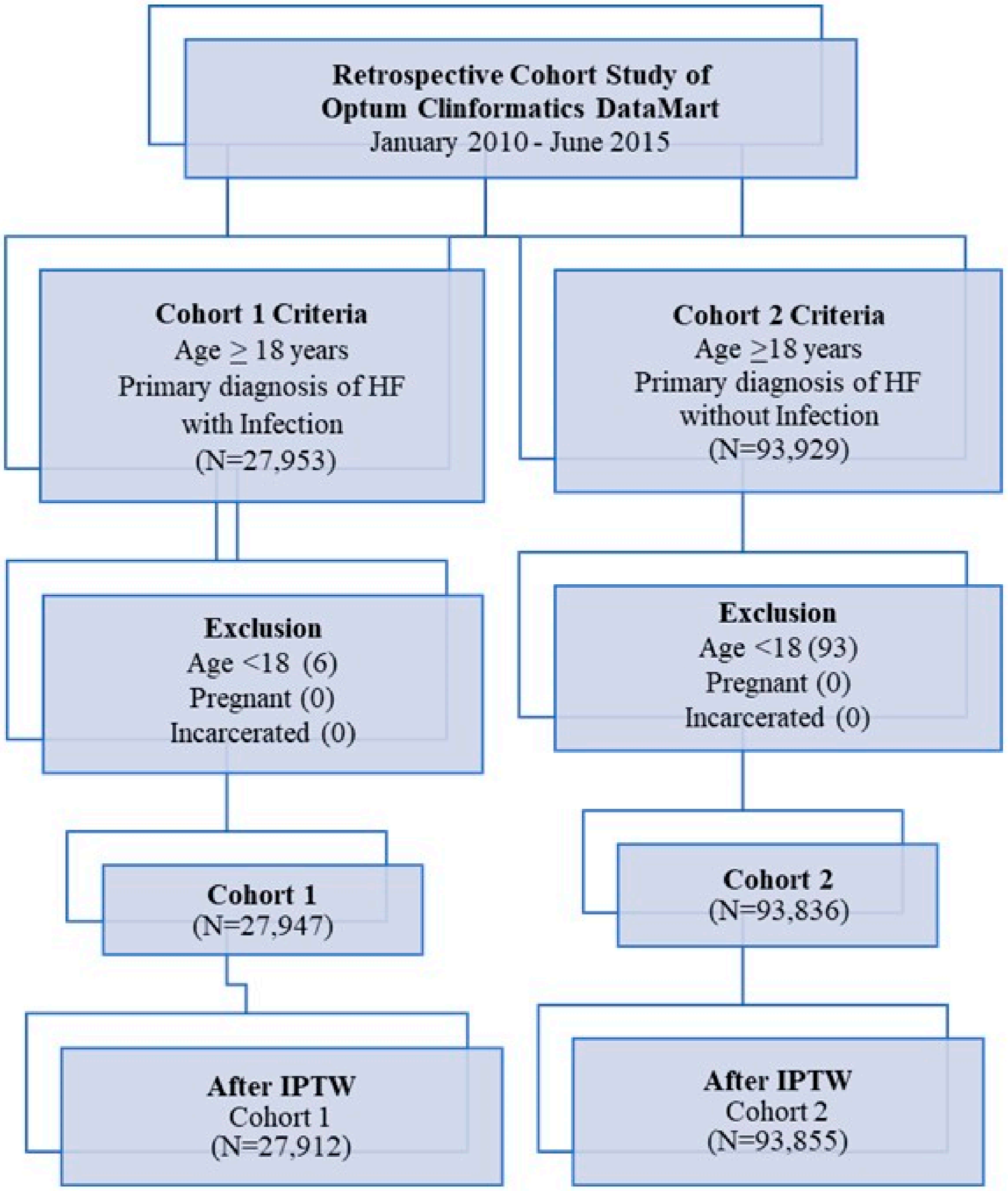

Figure S1. Assessment for Study Eligibility: Diagram of eligibility criteria for inclusion into (A) heart failure with infection or (B) heart failure without infection cohorts.

Table S1. Inverse Probability of Treatment Weighted Primary and Secondary Outcomes, and Multivariable Regression based on Pneumonia and Urinary Tract Infection Subgroups.

	Pneumonia	HF without Infection	Odds Ratio	95% CI	*p*-value
30-day readmission, (%)	16.5	15.7	1.06	1.02–1.11	0.0009
180-day readmission, (%)	38.4	38.4	0.99	0.96–1.03	0.923
30-day mortality, (%)	5.5	4.2	1.35	1.24–1.46	<0.001
180-day mortality, (%)	10.4	9.2	1.15	1.09–1.22	<0.001
ICU admission, (%)	38.6	33.9	1.24	1.19–1.28	<0.001
Length of stay, days (SD)	7.1 (6.5)	5.7 (5.7)	--	--	<0.001

Hospital charge, USD (SD)	63,188 (112,849)	50,943 (132,531)	--	--	<0.001
	**UTI**	**HF without Infection**			
30-day readmission, (%)	18.0	15.7	1.18	1.11–1.26	<0.001
180-day readmission, (%)	41.4	38.4	1.14	1.08–1.19	<0.001
30-day mortality, (%)	5.2	4.0	1.31	1.17–1.46	<0.001
180-day mortality, (%)	10.8	9.0	1.21	1.12–1.31	<0.001
ICU admission, (%)	33.7	33.8	0.99	0.95–1.04	0.771
Length of stay, days (SD)	6.4 (5.5)	5.7 (5.7)	--	--	<0.001
Hospital charge, USD (SD)	51,277 (92,570)	50,578 (132,067)	--	--	0.639

Table S2. Predictors of Acute Infection in Patients with Heart Failure.

Variable	Odds Ratio	95% Confidence Interval	*p*-value
Age	1.02	1.02–1.02	<0.0001
Female	1.36	1.32–1.39	<0.0001
Obesity	0.94	0.90–0.98	0.0020
Elixhauser comorbidity score	0.98	0.98–0.98	<0.0001
Ischemic heart disease	0.76	0.73–0.78	<0.0001
Acute renal failure	1.34	1.30–1.38	<0.0001
Anemia	1.11	1.08–1.14	<0.0001
Statin therapy	0.96	0.93–0.99	0.0042
